# Mobile Technologies for Every Library

**DOI:** 10.5195/jmla.2017.269

**Published:** 2017-07-01

**Authors:** Danielle Becker

I open this book review with a quote from the *New York Times* article, “Do We Really Need Negative Book Reviews?,” published on February 11, 2014, by Francine Prose and Zoe Heller [1]. Heller writes, “Banning ‘negativity’ is not just bad for the culture; it is unfair to authors. A review, however aggressively unfavorable, is generally obliged to provide supporting evidence for its judgments.” That said, there is abundant evidence to support my negative opinion of *Mobile Technologies for Every Library.*

The book’s author, Ann Whitney Gleason, AHIP, is more than qualified to write on the subject, given that her vast experience includes director for resources and systems, educational technology specialist, technology director, and chief information officer (CIO). This book’s shortcomings are in its timeliness and lack of insight into emerging technologies.

Like the author, my research interests are educational technologies in libraries, specifically mobile technologies, so when I had the opportunity to review this book, I was excited to learn something new. Admittedly, having written a fair amount on technology, I acknowledge how difficult it is to remain current. Some books, like Steve Krug’s *Don’t Make Me Think, Revisited: A Common Sense Approach to Web Usability,* retain usefulness. I found a great deal of value in the 2005 edition (and still do), even though the latest edition was published in 2014 (New Riders; ISBN: 978-0-321-96551-6). Some topics, like writing for the web, usability, and information architecture do not need to be updated every year because the overall concepts do not necessarily change greatly. But I think if authors are going to write about specific devices, apps, and emerging technologies, their objective is get their books in their readers’ hands quickly before the books go stale.

For the uninitiated reader, the inclusion of notes, further readings, and resources at the end of each chapter of *Mobile Technologies for Every Library* is helpful jumping-off points for beginning research or familiarizing oneself with the material. By the author’s own admission, her audience is someone who is wondering how to get started adopting mobile technology (p. vii). One of my first criticisms is why the first chapter included the history of mobile technology, complete with a simplistic illustration of the “brick” type of cell phone. I do not think the reader who wants to learn how (and why) to begin a mobile technology initiative would need this information. What difference does it make what cell phones were in use in the 1980s?

Chapter two gives the reader a broad overview of mobile devices, which is useful when first thinking about designing and implementing a mobile presence. These descriptions are compact, but the market share information is outdated, and the pie chart tallying 2013 world smartphone market shares (p. 13) has illegibly miniscule type, and the nuanced shades of gray are impossible to decipher. Chapter three, by far the most helpful chapter in this book, starts with a goal, asserting it will guide the reader through some of the “options, best practices, tips, and tools” (p. 25). It gives steps for preparing for a mobile presence, creating a mobile strategy, designing a mobile-friendly web presence, selecting content to include, and testing usability. The only thing I would take issue with is the brevity of the list of resources that are included in the testing section at the end of the book.

Chapters four to eight discuss mobile apps, mobile information seeking, best practices of mobile technology uses in libraries, and use of mobile technology in education. These general overviews are helpful for those readers who are completely new to the subject area. Fortunately, chapter four provided me with a bit of chuckle when I saw that Figure 4.2, “Android versions currently in use,” was three versions behind (Lollipop, Marshmallow, and Nougat are the three most current versions in use).

Chapter nine opens with a Wikipedia definition of “library out-reach” and moves through some worthwhile information, such as how librarians “must proactively seek integration into teaching and learning, as well as into community activities, in order to provide value and return on investment or risk losing funding in these competitive budgetary times” (p. 99). Chapter ten closes the book with a look into the future of mobile technology. Sadly, this chapter is woefully out of date (e.g., 2014 Horizon report, Eric Topol’s 2009 TED Talk, a “recent” (2012) article in *Scientific American,* and so on), and an overview of “wearable technology” describes devices like the Pebble watch and FitBit but does not mention the wildly popular GoPro camera and nothing about the usefulness of these devices in libraries. Furthermore, virtual reality (VR) and augmented reality (AR) are not new and exciting, nor is Google Glass, for that matter.

I do not recommend this book to any librarian looking for an affordable book that thoroughly covers the topic of mobile technologies in libraries. Instead, look to Ben Rawlins’ *Mobile Technologies in Libraries* (Rowman & Littlefield; 2016; ISBN: 978-1-4422-6423-6). As Prose asserts in the previously mentioned *New York Times* article, “Needless to say, criticism is a matter of opinion,” and this review, although critical, is my opinion.
